# Functional Characterization of Mung Bean Meal Protein-Derived Antioxidant Peptides

**DOI:** 10.3390/molecules26061515

**Published:** 2021-03-10

**Authors:** Chanikan Sonklin, Adeola M. Alashi, Natta Laohakunjit, Rotimi E. Aluko

**Affiliations:** 1 Department of Industrial Chemistry, Faculty of Applied Science, King Mongkut’s University of Technology North Bangkok, 1518 Pracharat 1 Rd., Wongsawang, Bangsue, Bangkok 10800, Thailand; chanikan.s@sci.kmutnb.ac.th; 2 Department of Food and Human Nutritional Sciences, University of Manitoba, Winnipeg, MB R3T 2N2, Canada; monisola.alashi@umanitoba.ca; 3 School of Bioresources and Technology, King Mongkut’s University of Technology Thonburi, 49 Tein-talay 25 Rd., Tha-kam, Bangkhuntein, Bangkok 10150, Thailand; nutta.lao@kmutt.ac.th; 4 Richardson Centre for Functional Foods and Nutraceuticals, University of Manitoba, Winnipeg, MB R3T 2N2, Canada

**Keywords:** mung bean, bromelain, membrane ultrafiltration, protein hydrolysate, antioxidant, peptide sequencing, RP-HPLC

## Abstract

The aim of this work was to characterize the antioxidant properties of some of the peptides present in bromelain mung bean meal protein hydrolysate (MMPH). The MMPH was subjected to two rounds of bioassay-guided reversed-phase HPLC separation followed by peptide identification in the most potent fractions using tandem mass spectrometry. Twelve antioxidant peptides, namely, HC, CGN, LAN, CTN, LAF, CSGD, MMGW, QFAAD, ERF, EYW, FLQL, and QFAW were identified and assayed for antioxidant properties. CTN, HC, CGN, and CSGD were the most potent (*p* < 0.05) DPPH radical scavengers with EC_50_ values of 0.30, 0.29, 0.28, and 0.30 mg/mL, respectively, which are lower than the 0.03 mg/mL obtained for reduced glutathione (GSH). CTN, HC, CGN, and CSGD exhibited the most potent (*p* < 0.05) scavenging activities against hydroxyl and superoxide radicals with EC_50_ values that are similar to those of GSH. The cysteine-containing peptides also had stronger ferric reducing antioxidant power and metal chelation activity than peptides devoid of cysteine. In contrast, MMGW, ERF, and EYW had poor radical scavenging and metal chelation activities. We conclude that the availability of the sulfhydryl group may have enhanced antioxidant potency while the presence of bulky groups such phenylalanine and tryptophan had an opposite effect.

## 1. Introduction

Free radicals, especially reactive oxygen species such as superoxide radicals (O_2_^•^), hydroxyl radicals (OH^•^), peroxyl radicals (ROO^•^), hydrogen peroxide (H_2_O_2_), and singlet oxygen, are critical because they directly affect cells of living organisms, which, if unattended to, can lead to health problems [1]. This is because only micrograms or lower quantities of ROS can play beneficial roles in the human body while excessive levels could become toxic and lead to cellular damages. Excessive levels of ROS can arise if there is an imbalance between their production and the natural detoxification processes in biological systems. The presence of excess ROS in the body can lead to oxidative stress, which damages vital macromolecules (especially proteins and lipids), and lead to accelerated aging as well as increased risk of developing various diseases, like Parkinson’s, Alzheimer’s, atherosclerosis, diabetes, cancer, and neurodegenerative disease [2,3,4,5].

Antioxidant substances, both synthetic and natural have become significant for suppressing reactive species to maintain human health. However, synthetic antioxidants like butylated hydroxyanisole (BHA) and butylated hydroxytoluene (BHT) may have carcinogenic side effects and their uses are being discouraged [6]. Therefore, several natural compounds are being studied as potential replacements for synthetic antioxidants. In order to be acceptable alternatives, these compounds should be able to neutralize ROS and prevent or reduce cellular damages. One of the most studied natural alternatives to synthetic antioxidants are the food protein-derived peptides, which are normally obtained through enzymatic hydrolysis of food proteins and have been extensively evaluated for potential therapeutic functions in preventing cellular damages from oxidative stress and promoting human health [7]. Bioactive peptides normally contain 2–20 amino acid residues with low molecular weight and have a variety of biological activities depending on the type of amino acid, molecular size, and sequence [8,9]. The antioxidant activities of several plant protein-derived peptides have been determined including chickpea [10,11], wheat [12], barley [13], soybean [14], corn [15], rapeseed [16,17], and mung bean [18,19,20,21].

Mung bean meal is a by-product from vermicelli processing and is usually discarded as waste even though it has approximately 70% protein (*w*/*w* dry basis). In a previous study, mung bean meal was produced as a flavor enhancer using enzymatic hydrolysis with bromelain [22]. Peptides and free amino acid in the hydrolysate are key compounds for its flavor and taste. However, these peptide and amino acids could play various functional properties and bioactive activities, including the ability to act as antioxidants. Previous studies have reported other bioactive properties in mung bean peptides and protein hydrolysates such as antihypertensive [23,24], anti-allergic [25], and anti-tumor [26]. However, only few studies have reported the antioxidant activities of specific peptide sequences derived from mung bean protein hydrolysate. Moreover, research exploring the active sequences of the antioxidant peptides obtained from mung bean protein hydrolysate using bromelain is limited. Knowledge of amino acid sequence is critical to elucidating structure-function properties with a view to developing highly potent antioxidant peptides. Therefore, this study was carried out to identify and determine the in vitro functional performance of antioxidant peptide sequences present in a bromelain mung bean protein hydrolysate.

## 2. Results and Discussion

### 2.1. Antioxidant Activities of Peptide Fractions from the First Round of RP(C12)-HPLC

**Figure 1:**
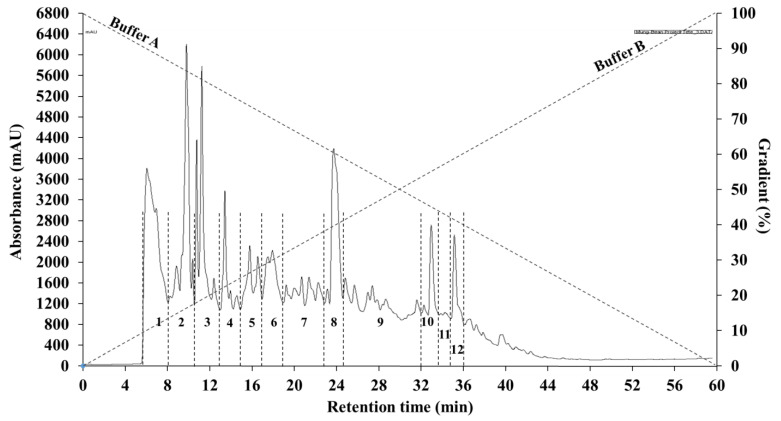
Initial separation of mung bean protein hydrolysate by reversed-phase (C12) HPLC into 12 fractions (1–12). Reprinted from *Journal of Functional Foods*, Vol. 64, Sonklin, C., Alashi, A.M., Laohakunjit, L., Kerdchoechuen, O., & Aluko, R.E. Identification of antihypertensive peptides from mung bean protein hydrolysate and their effects in spontaneously hypertensive rats, Article 103635. Copyright (2020), with permission from Elsevier.

Peptides present in the <1 kDa fraction of mung bean protein hydrolysate that contributed to a high antioxidant activity potential were identified using two consecutive rounds of RP(C12)HPLC as previously described [23]. In the first RP(C12)HPLC round, the mung bean peptides were pooled into twelve fractions (1–12) based on their retention time (Figure 1).

**Figure 2:**
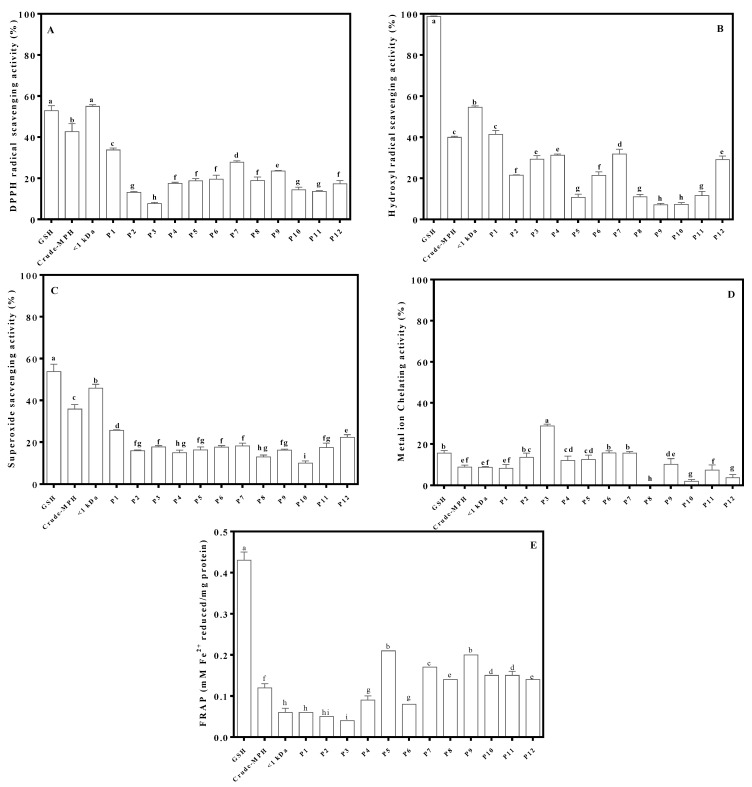
Antioxidant activities of crude mung bean meal protein hydrolysate (MMPH), <1 kDa peptides and peptide fractions derived from the 1st round of RP(C12)HPLC (P1–P10): DPPH scavenging activity (**A**), hydroxyl radical scavenging activity (**B**), superoxide radical scavenging activity (**C**), FRAP (**D**), and metal ion chelation activity (**E**). Data are expressed as mean ± standard deviation (*n* = 3). a,b,c,… Different letters indicate significantly different mean values (*p* < 0.05).

The principle of the RP-HPLC involves separating peptides based on their differences in hydrophobicity (hydrophobic/hydrophilic characters). The hydrophilic peptides were eluted first while the more hydrophobic peptides become eluted later. The twelve peptide fractions were assayed for their antioxidant activities using five different methods including DPPH radical scavenging activity, hydroxyl radical scavenging activity, superoxide radical scavenging activity, FRAP, and metal chelating activity (Figure 2).

The results indicate that fraction P1 had the highest DPPH scavenging, hydroxyl, and superoxide radical scavenging activities at 34.92%, 41.15%, and 25.63% respectively. Fractions P5 and P9 had the strongest FRAP with 0.21 and 0.20 mM Fe^2+^ reduced/mg peptide, respectively, while fraction 3 showed the highest metal chelation activity at 28.79%. The results suggest that most of the fractions contributed to at least one antioxidant activity, which reflect the presence of several antioxidant peptides with a wide range of hydrophobicity (directly related to elution time) in the mung bean protein hydrolysate. Most of the later-eluting fractions (P5–P12) showed a high potential for antioxidant activities, except for metal chelation. Fraction P1 had the best antioxidant activities for DPPH radical, hydroxyl radical and superoxide radical but poor activity for FRAP and metal ion chelation. Therefore, the results revealed that not only later-eluting (higher hydrophobic character) peptide fractions have strong antioxidant activities. This might have been influenced by the high hydrophilic character of fraction P1, which indicate presence of excess electrons available for neutralizing free radicals. The results are consistent with an earlier report suggesting that the presence of aspartic acid residues enhance DPPH radical scavenging activity of food protein hydrolysates [27]. Fraction P1 was selected for further separation by a second round of RP(C12)HPLC and subsequent peptide sequence identification because of the strong free radical scavenging activities.

### 2.2. Antioxidant Activities of Peptide Fractions from the Second Round of RP(C12)HPLC

**Figure 3:**
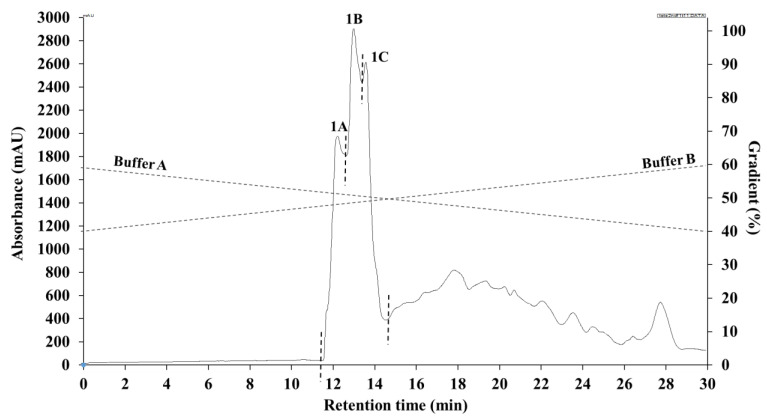
Separation of fraction P1 by second round of reversed-phase (C12) HPLC into 1A, 1B, and 1C sub-fractions.

In order to identify the peptides potentially responsible for the antioxidant activities, fraction P1 was further separated by a second RP(C12)HPLC and pooled into three fractions (1A, 1B, and 1C) as shown in Figure 3.

**Figure 4:**
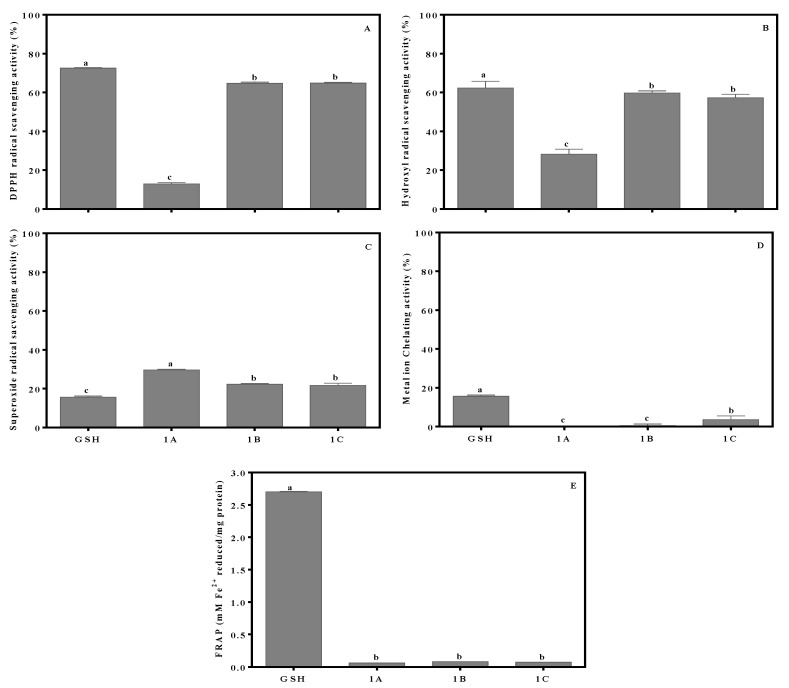
Antioxidant activities of P1 fractions derived from the second round of RP(C12)HPLC (1A–1C): DPPH scavenging activity (**A**), hydroxyl radical scavenging activity (**B**), superoxide radical scavenging activity (**C**), metal ion chelation activity (**D**), and FRAP (**E**). Data are expressed as mean ± standard deviation (*n* = 3). a,b,c Different letters indicate significantly different mean values (*p* < 0.05).

Results of the antioxidant assays indicate similarities in potency of fraction 1B and 1C, which were different from that of fraction 1A (Figure 4). For example, fractions 1B and 1C had DPPH radical scavenging activities of 64.70% and 64.50%, in addition to hydroxyl radical scavenging activity of 59.78% and 57.30%, respectively. However, the superoxide radical scavenging activity was lower at 22.27% and 21.70% for fractions 1B and 1C, respectively. In contrast, fractions 1A, 1B, and 1C all had very poor FRAP and metal ion chelation activity. Based on their strong radical scavenging activities, fractions 1B and 1C were selected for peptide sequencing by tandem mass spectrometry (MS/MS).

### 2.3. Sequencing and Antioxidant Activities of Peptides

**Figure 5:**
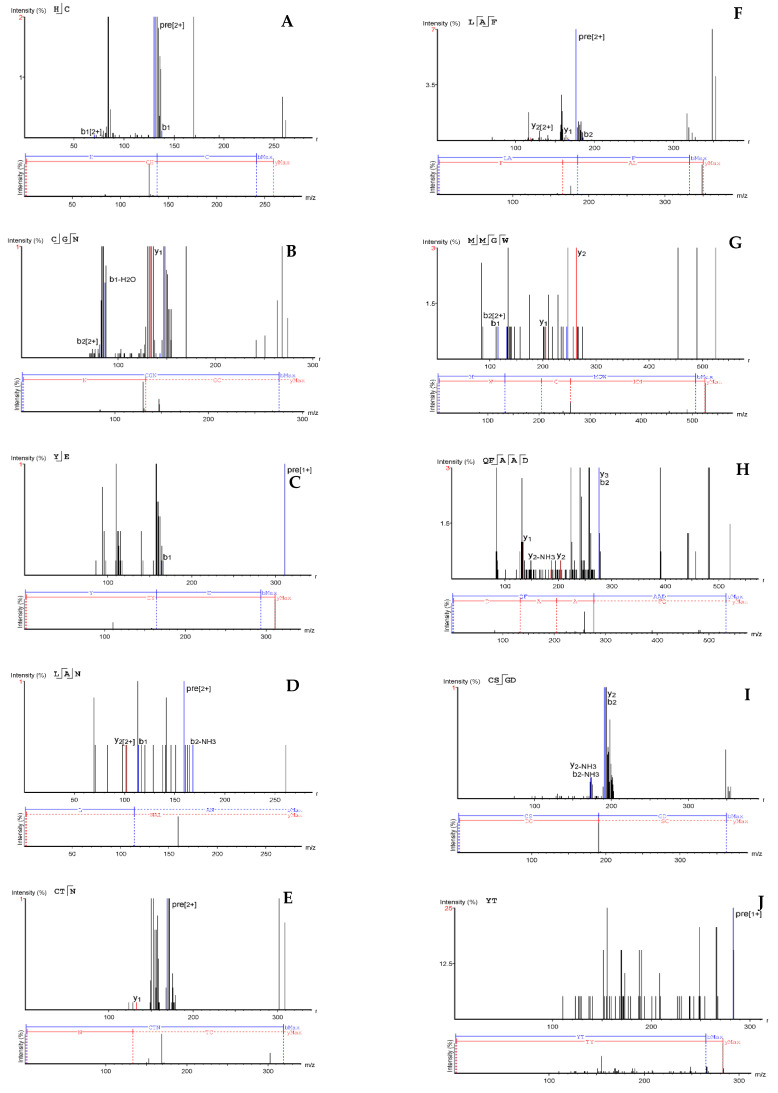
De-convoluted annotated MS/MS fragmentation spectra of peptides in fraction 1B and the corresponding de novo sequencing: HC (**A**), CGN (**B**), YE (**C**), LAN (**D**), CTN (**E**), LAF (**F**), MMGW (**G**), QFAAD (**H**), CSGD (**I**), and YT (**J**).

**Figure 6:**
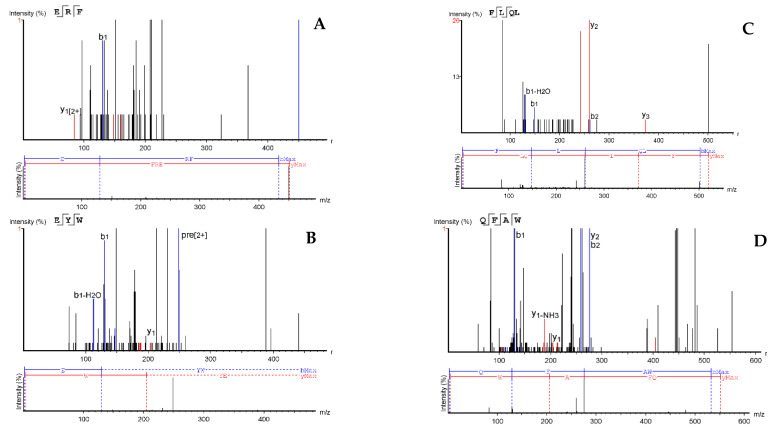
De-convoluted annotated MS/MS fragmentation spectra of peptides in fraction 1C and the corresponding de novo sequencing: ERF (**A**), EYW (**B**), FLQL (**C**), and QFAW (**D**).

A precursor/fragment ion relationship is established for target amino acid sequence, which identifies each peptide at a selected mass-to-charge ratio (*m*/*z*) value that can be detected using a mass spectrometer. From the deconvoluted diagrams shown in Figure 5 and Figure 6, 14 y-ions were detected from 1B and 9 from 1C. The MS/MS ions and derived amino acid sequences for peptides present in fractions 1B and 1C are shown in Figure 5 and Figure 6, respectively. However, it should be noted that de novo peptide sequencing has limitations in the absence of a protein database to confirm the obtained sequences.

The ten oligopeptides obtained from fraction 1B are HC, YT, YE, LAN, CTN, LAF, CGN, CSGD, MMGW, and QFAAD while those present in fraction 1C are ERF, EYW, FLQL, and QFAW. The twelve peptide sequences (HC, CGN, LAN, CTN, LAF, CSGD, MMGW, QFAAD, ERF, EYW, FLQL, and QFAW) having the highest intensity from the mass spectrometry were selected for synthesis and their antioxidant properties determined as shown in Table 1. Leucine and isoleucine have similar MS/MS fragmentation patterns but leucine was selected for the peptides because our previous work showed about 1.5 times the level of isoleucine in the protein hydrolysate [18]. However, due to the potential for high false positive rate during de novo sequencing, a manual validation of the presence of all the peptides will have to be conducted in future studies.

#### 2.3.1. DPPH Radical Scavenging Activity (DRSA)

The peptides CGN, HC, CTN, and CSGD had the strongest DRSA as reflected in the lower EC_50_ values when compared to the remaining peptides (Table 1). The results are consistent with a previous study, which indicated that the presence of asparagine (*N*), threonine (*T*), and aspartic acid (*D*) contributes strongly to the DRSA of food protein hydrolysates [27].

The presence of excess or readily donated electrons on these amino acid residues could have potentiated the DRSA of CGN, HC, CTN, and CSGD. Moreover, cysteine has been reported to enhance DRSA because the sulfur molecule and sulfhydryl group (SH group) in its molecule are stronger oxidizing agents than other amino acids without sulfur in their structure. This is more effective especially if the Cys is located in the N- and C-terminal [9,15], which supports the strong DRSA of CGN, HC, CTN, and CSGD. The SH group can donate hydrogen atom to quench the DPPH radical, and in the process becomes S·, which reacts with and neutralizes other DPPH radical molecules to become sulfonyl (SO_2_) or disulfide (S-S) compounds, resulting in free radical chain termination [28]. The results obtained in this work show that all the most active peptides (CGN, HC, CTN, and CSGD) contained Cys in their N-terminal or C-terminal. The results are consistent with the role of cysteine as a major contributing amino acid to the strong antioxidant potency of GSH, a natural cellular tripeptide [9,29]. While the peptides obtained in this study had higher EC_50_ value of DRSA than GSH, the EC_50_ values of CGN, HC, CTN, and CSGD are lower than that of GPP (EC_50_ of 1.927 mg/mL), a peptide derived from Bluefin leatherjacket [30]. The lower EC_50_ value indicates higher antioxidant potential. The results indicated that these peptides could inhibit DPPH radical better than GPP peptide. In contrast, peptide ERF had the poorest DRSA with a high EC_50_ value of 4.87 mg/mL, which is almost 162 times lower than the activity obtained for GSH, the potent cellular antioxidant peptide. EYW, which has glutamic acid (*E*) at the N-terminal and QFAW also showed weaker DRSA when compared to CGN, HC, CTN, and CSGD. The presence of lysine (*Y*) has been reported to be negatively correlated with DRSA, which could be responsible for the weaker activity of EYW. It is pertinent to note that EYW, QFAW, and MMGW all have tryptophan at the C-terminal, which seem to reduce DRSA potency of the peptides. Peptide LAN had poor DPPH activities and were not able to effectively scavenge DPPH. Similarly, the three peptides with no detectable activity (LAF, QFAAD, and FLQL) all contain phenylalanine but whether this residue is responsible for the lack of DRSA remains to be investigated. However, for this set of peptides, the presence of aromatic amino acids correlated with weak DRSA. The results are consistent with previous studies that suggested that, while the low molecular weight of peptides could promote antioxidant activity, the sequence within the peptide chain and the position and type of amino acids were the main factors [11,31,32].

#### 2.3.2. Hydroxyl Radical Scavenging Activity (HRSA)

The hydroxyl radical is one of the most reactive oxygen radicals, causing radical chain reactions and inducing severe damage to cells in the human body, including destruction of carbohydrates, nucleic acids, lipids, and proteins, which makes it an important free radical and a target of antioxidants in the human body [29,33]. Peptides LAF, HC, CTN, CGN, and CSGD were the most potent hydroxyl radical scavengers with EC_50_ values lower than 1 mg/mL and similar to that of GSH (Table 1). Other peptides, ERF, QFAAD, MMGW, and LAN, showed poor hydroxyl scavenging radical activity with higher EC_50_ values while EYW, FLQL, and QFAW had no detectable activities. A comparison of LAN with LAF suggests that the presence of phenylalanine (*F*) at the C-terminal provides better HRSA than asparagine in the same position. The observed difference may be due to the reported ability of the aromatic ring in phenylalanine to donate hydrogen atom to the hydroxyl radical and thus terminate the free radical chain reaction [32,33]. HC, CTN, CGN, and CSGD exhibited strong hydroxyl radical scavenging activity that is similar to their DRSA, which could be attributed to the presence of cysteine at the N- or C-terminal. The sulfhydryl group in cysteine can also donate a proton to neutralize the hydroxyl radical. Therefore, the results suggest that the presence of cysteine or phenylalanine at the N- or C-terminal could make a significant contribution to the HRSA of peptides [32]. The EC_50_ values for LAF, HC, CTN, CGN, and CSGD are lower (stronger HRSA) than the 2.358 mg/mL reported for Bluefin leather jacket peptide GPP [30] while the LAF had similar value as VLYEE (EC_50_ 0.353 mg/mL), a peptide obtained from croceine muscle protein hydrolysate [29] and FY (EC_50_ 0.41 mg/mL), a peptide derived from perilla seed protein [34]. In the case of phenylalanine, which is present in LAF and FY (perilla seed peptide), both of these possessed strong HRSA. On the other hand, leucine, which is found in LAF and YL (perilla seed peptide) lacked HRSA. The results suggest that the presence of phenylalanine at the N- or C-terminal could be largely related effective HRSA. In contrast, peptides that contained leucine at C-terminal had low HRSA. Therefore, the results support the principle that type of amino acid and its position has significant effect on antioxidant activity.

#### 2.3.3. Superoxide Radical Scavenging Activity (SRSA)

Another important oxygen species or free radical is the superoxide radical. The strongest SRSA (lowest EC_50_ value) was also displayed by the peptide LAF and is similar to the GSH activity (Table 1). Other peptides with strong EC_50_ values (<1.0 mg/mL) include HC, CGN, CTN, CSGD, and QFAAD, with activities that are similar to that of GSH. The peptides FLQL and QFAW did not have any measurable SRSA. Apart from LAF, the presence of cysteine may have also contributed to the strong SRSA of some of the peptides. Therefore, cysteine might be the most important amino acid responsible for scavenging of the superoxide radicals in this study. Jin et al. [15] also reported that the corn protein-derived peptide CSQAPLA with a cysteine residue at the N-terminal had better SRSA than two other peptides that contained no cysteine. Both the free forms and residual side chains of cysteine can oxidize the oxygen free radical (oxygen free radical-mediated oxidation) to becomes cysteine disulfides or sulfonic acid, which results in the termination of the free radical reaction [35]. The peptide CYIE obtained from protein hydrolysates of blood cockle, which had cysteine at its N-terminal, had an SRSA EC_50_ value of 2.31 mg/mL [36], which is higher (weaker activity) than those of HC, CGN, CTN, CSGD, and QFAAD in the present study. Previous reports have also reported strong contributions of cysteine to the SRSA of CSQAPLA, CQV, QCV, QVC, and QCA [15,37]. Moreover, the peptides LAF, CGN, CTN, and CSGD all exhibited stronger superoxide radicals scavenging activity than GVPLT (EC_50_ 2.881 mg/mL) obtained from Bluefin leather jacket [30], and MDLFTE (EC_50_ 0.75 mg/mL) and WPPD (EC_50_ 0.46 mg/mL) from hydrolysates of blood cockle [36].

#### 2.3.4. Ferric Reducing Antioxidant Power (FRAP)

Only the peptide HC showed strong FRAP with a similar value as the standard GSH while CSGD and CTN had moderate values (Table 1). Interestingly, all the peptides with the strong or moderate FRAP also had cysteine in their sequence. The presence of cysteine at the peptide terminals may have facilitated electron donation to reduce the ferric ion to the more stable divalent ferrous ion. The results are consistent with a previous report indicating strong contributions of cysteine to FRAP of peptides [15,27]. The presence of histidine might also be another factor that contributed positively to the strong FRAP of HC. Usually, histidine-containing peptides act as antioxidants mainly by electron transfer, which could assist in reducing the ferric ion, depending on the pH of the environment [38]. Furthermore, the ability of the imidazole group to undergo resonance stabilization can enhance electron sharing or donation to the ferric ion [38]. In contrast, peptides such as ERF, EYW, FLQL, QFAAD, and LAF that are devoid of cysteine or histidine residues had poor ability to share or donate electrons to the ferric ion, which could be responsible for the observed poor FRAP. While glutamic acid (*E*) has been suggested to contribute to a strong FRAP of peptides, the presence of aromatic amino acids is detrimental and may have contributed to the weak FRAP of ERF and EYW [27].

#### 2.3.5. Metal Ion Chelating Activity (MCA)

Metal chelation is necessary to protect living cells from the toxic effects of metal-catalyzed free radical generating reactions, especially through the Fenton reaction. The MCA refers to the capacity of peptides to bind metal ions such as Cu^2+^ and Fe^2+^ thereby preventing the oxidative damage of biological macromolecules in human body such as proteins and nucleic acids [8]. Mung bean peptides had variable MCA with stronger activities (EC_50_ values less than the 0.18 mg/mL for GSH) obtained for CGN, CTN, and QFAW (Table 1). The peptides CSGD and LAN had slightly higher EC_50_ values than GSH but are stronger metal chelators when compared to HC, MMGW, QFAAD, ERF, and EYW. While previous works have suggested that amino acids like sulfur and acidic amino acids might have significant effects on the MCA of peptide, results from the present study indicate the importance of other amino acid residues. The suggested contributions of sulfur and acidic amino acids are due to the excess electrons, which could enhance electrostatic and ionic interactions with iron [39]. However, with the exception of LAN and QFAAD, the present results differ from previous reports showing that chickpea protein hydrolysate [40] and rice bran protein hydrolysate [41] with strong MCA contained asparagine and glutamine. However, chickpea peptides containing asparagine with same -CONH_2_ group as glutamine (*Q*) at the N-terminal position of peptides also showed very strong MCA [40], which is similar to the results obtained for QFAW in this research. Therefore, it is possible that the -CONH_2_ group in its side chain molecule can chelate the transition metal ions (Fe^2+^ and Cu^2+^). In contrast, FLQL with glutamine in the penultimate position had no detectable MCA, which indicates that position of the amino acid is critical to bioactive properties. The MCA of glutamine is believed to be due to the univalent -CONH_2_ group in its structure. The carbonyl group (C=O) acts as a ligand molecule, and can tightly bind with metal ions to becomes a stable complex molecule [42]. High MCA is not only related to sulfur and acidic amino acids but also basic amino acids. Peptide HC had moderate MCA probably because the imidazole ring in histidine side chain can interact with metal ions [40]. Therefore, sulfur, acidic and basic amino acids had significant positive effects on MCA of the peptides.

## 3. Materials and Methods

### 3.1. Materials

Mung bean meal was provided by Sittinan Co. Ltd., Lat Lum Kaeo District, Pathum Thani, Thailand. Before hydrolysis, mung bean meal was extracted twice with hexane to obtain the defatted meal. Bromelain was provided by KMuch-Industry Co., Ltd. (Bangkok, Thailand). All other chemical reagents were of analytical grade and purchased from Fisher Scientific (Oakville, ON, Canada) or Sigma Aldrich (St. Louis, MO, USA).

### 3.2. Preparation of Peptide Fractions from Mung Bean Hydrolysate

Defatted mung bean meal was hydrolyzed using 15% (*w*/*w*) bromelain (enzyme activity = 97,540 CDU) at pH 6.0 and temperature 50 °C for 12 h according to a previously detailed method [43]. The mung bean meal protein hydrolysate (MMPH) was further fractionated using an ultrafiltration membrane with molecular weight cut off 1 kDa. The permeate (<1 kDa peptide fraction) was collected, freeze dried, and stored at −20 °C.

### 3.3. First Round of Reversed-Phase High-Performance Liquid Chromatography (RP(C12)HPLC) Peptide Separation

The less than 1 kDa peptide fraction was further purified by a two-step RP(C12)HPLC using a Varian 940-LC system (Agilent Technologies, Santa Clara, CA, USA) according to a previous method [23]. Briefly, the <1 kDa peptide fraction was diluted in Buffer A (0.1% trifluoroacetic acid, TFA in Milli-Q water) at a concentration of 100 mg/mL and was filtered sequentially first through a 0.45 μm and then 0.2 μm membrane discs before injection. The filtered sample was injected (4 mL) onto a 21 × 250 mm (5 μm) C12 preparative column (Phenomenex Inc., Torrance, CA, USA) and Buffer B (0.1% TFA in methanol) was used to elute peptides at a flow rate of 10 mL/min using a linear gradient from 0%–100% Buffer B over 60 min. Eluted peptides were detected at 214 nm, collected every minute and pooled into 12 fractions (P1–P12) based on their elution time. The 12 fractions were evaporated under vacuum in a rotary evaporator (maintained at a temperature at 45 °C) in order to remove solvent from the fractions; the aqueous residues were then freeze-dried and analyzed for antioxidant activities.

### 3.4. Second Round of RP(C12)HPLC Separation

Fraction (P1) from the initial RP(C12)HPLC was further purified on the C12 preparative column since it showed the highest antioxidant activities. Fraction P1 was dissolved in Buffer A at a concentration of 25 mg/mL. The sample was filtered through a 0.2 µm disc filter and 2 mL injected onto the preparative C12 column. Sample elution was carried out at a flowrate of 2 mL/min with a linear gradient of 40–60% Buffer B for 45 min; eluted peptides were detected at 214 nm and collected every minute. P1 was pooled into 3 fractions (1A–C) based on elution time. The fractions obtained were evaporated under vacuum in a rotary evaporator as described above, and the aqueous residues were freeze-dried and stored at −20 °C. Antioxidant activity of the freeze-dried fractions were determined.

### 3.5. Identification of Peptide Sequences

Amino acid sequence of peptides present in 1B and 1C (fractions with strongest antioxidant activities) were carried out by first generating the tandem mass spectrometer (MS/MS) spectra using an Absciex QTRAP^®^ 6500 MS System coupled with an electrospray ionization source (Absciex, Foster City, CA, USA) as previously described [23]. The freeze-dried fractions were dissolved (100 µg/mL) in 20% acetonitrile followed by direct infusion (10 µL) into the mass spectrometer. The following instrument parameters were used: Ion source: Turbo Spray Ion Drive, Operation mass scan mode: Low mass mode with mass scan rage 55–1,000 Da, Vacuum gauge: 3.1 × 10^−5^ Torr, Source temperature (at set point): 399 °C, Scan Type: Enhanced Product Ion (EPI), Polarity: Positive ion mode and Scan rate: 2000 Da/s. The MS/MS spectra data were obtained from the Absciex operating program. The amino acid sequence of each peptide was subsequently identified by de novo peptide sequencing using the PEAKS^®^ software, version 7 (Bioinformatics Solutions Inc., Waterloo, ON, Canada). The raw files from Absciex operating program (.wiff) were loaded into PEAKS operating program. The high intensity peaks from 1B and 1C were selected for sequence identification. The condition of sequence identity was set with trypsin enzyme specificity (instead of bromelain), precursor mass tolerance was set to 0.5 Da, fragment ion tolerance was set to 0.5 Da and with acceptable average local confidence (ALC) ≥75%. The predicted peptide sequences were synthesized (>95% purity) by Genscript USA Inc. (Genscript USA Inc., Piscataway, NJ, USA) and their antioxidant activities analyzed.

### 3.6. Determination of Antioxidant Properties

#### 3.6.1. DPPH Radical Scavenging Activity

The scavenging activity of peptide samples (0.6 mg/mL) against DPPH radical was carried out according to the method described by Alashi et al. [44]. Peptide samples were dissolved in 0.1 M sodium phosphate buffer, pH 7.0 containing 1% (*w*/*v*) Triton X-100. DPPH was dissolved in 95% ethanol to a final concentration of 100 μM. Peptide samples (100 μL) at various concentrations were mixed with 100 μL of the DPPH solution in a 96-well plate while the blank contained 100 μL buffer instead of sample. The reaction mixtures were subsequently incubated at room temperature in the dark for 30 min followed by measurement of absorbance values at 517 nm. Reduced glutathione (GSH) was used as a positive control and assayed the same way as samples and blank.

The DPPH radical-scavenging activity was calculated using the following equation:

(1) $$\mathrm{DPPH}\;\mathrm{radical}\;\mathrm{scavenging}\;\mathrm{activity}\;\left(\%\right)=\frac{A_{\mathrm{blank}}-{\;A}_{\mathrm{sample}}}{A_{\mathrm{blank}}}\;\times 100$$

The effective peptide concentration that scavenged 50% of the DPPH radicals (EC_50_) was determined from the regression plot of concentration against percentage scavenging activity using GraphPad Prism version 6.0 (GraphPad Software, San Diego, CA, USA).

#### 3.6.2. Hydroxyl Radical Scavenging Activity

The hydroxyl radical scavenging assay was carried out using the modified method described by Ajibola et al. [45]. Peptide samples and positive control (GSH) were dissolved in 0.1 M sodium phosphate buffer (pH 7.4) at various concentrations. The reactions were carried out in a 96-well microplate, where 50 μL of samples or buffer (blank) was mixed with 50 μL of 3 mM 1,10-phenanthroline in 0.1 M sodium phosphate buffer (pH 7.4) and 50 μL of 3 mM FeSO_4_. To initiate the Fenton reaction, 50 μL of 0.01% hydrogen peroxide (H_2_O_2_) was added and absorbance read at 536 nm every 10 min for 1 h while incubating at 37 °C with continuous shaking. The percentage hydroxyl radical scavenging activity was calculated using the following equation:

(2) $$\mathrm{Hydroxyl}\;\mathrm{radical}\;\mathrm{scavenging}\;\mathrm{activity}\;\left(\%\right)=\;\frac{({\Delta \mathrm{Amin}}^{-1}\left(\mathrm{blank}\right)-{\;\Delta \mathrm{Amin}}^{-1}\left(\mathrm{sample}\right)\;}{{\Delta \mathrm{Amin}}^{-1}\left(\mathrm{blank}\right)}\;\times 100$$

where, ${\Delta \mathrm{Amin}}^{-1}\left(\mathrm{blank}\right)$ and ${\Delta \mathrm{Amin}}^{-1}\left(\mathrm{sample}\right)$ are the change in rate of reaction of blank and sample, respectively. EC_50_ values were calculated as described above for DPPH.

#### 3.6.3. Superoxide Radical Scavenging Activity

Samples at varying concentrations were prepared by dissolving in 50 mM Tris–HCl buffer, pH 8.3 containing 1 mM EDTA. Samples (80 µL) were pipetted into clear microplate wells while a similar volume of buffer was used for the blank well and GSH as the positive control. After adding 40 µL of 1.5 mM pyrogallol (prepared in 10 mM HCl), and 80 µL buffer to each well, the reaction was measured immediately using a spectrophotometer at 420 nm for 4 min at intervals of 1 min [46]. The superoxide scavenging activity was calculated using the following equation:

(3) $$\mathrm{Uperoxide}\;\mathrm{scavenging}\;\mathrm{activity}\;\left(\%\right)=\;\frac{({\Delta \mathrm{Amin}}^{-1}\left(\mathrm{blank}\right)-{\;\Delta \mathrm{Amin}}^{-1}\left(\mathrm{sample}\right)\;}{{\Delta \mathrm{Amin}}^{-1}\left(\mathrm{blank}\right)}\;\times 100$$

where, ${\Delta \mathrm{Amin}}^{-1}\left(\mathrm{blank}\right)$ and ${\Delta \mathrm{amin}}^{-1}\left(\mathrm{sample}\right)$ are the change in rate of reaction of blank and samples, respectively. EC_50_ values were calculated as described above for DPPH.

#### 3.6.4. Ferric Reducing Antioxidant Power (FRAP)

The FRAP assay was performed according to the modified method of Karamać et al. [47]. The FRAP reagent was prepared by mixing 0.3 M acetate buffer, 10 mM TPTZ at pH 3.6 in 40 mM HCl, and 20 mM FeCl_3_.6H_2_O pH 3.6 at ratio of 5:1:1 (*v*/*v*/*v*). Samples or GSH were dissolved in deionized water to obtain varying reaction concentrations and the reaction carried out in a 96-well microplate. The FRAP reagents were incubated at 37 °C and 200 µL taken and added to 40 µL of samples followed by absorbance measurement at 593 nm. A standard curve was generated using FeSO_4_·7H_2_O (0.03–0.9 µmol/mL) and results were reported as mmol of Fe^2+^ reduced per g protein from the regression slope of the standard curve.

#### 3.6.5. Metal Ion Chelating Activity

To assay for metal ion chelating activity, samples were prepared to varying concentrations in deionized water. Samples, GSH, or blank (water) were mixed separately with 0.05 mL of 2 mM FeCl_2_, 1.85 mL of deionized water, and 0.1 mL of 5 mM of ferrozine solution in a reaction tube for 10 min at room temperature. Subsequently, 200 µL of each sample solution were pipetted into 96 well microplates and absorbance measured at 562 nm [46]. The percentage metal ion chelating activity was calculated using the following equation:

(4) $$\mathrm{Metal}\;\mathrm{chelating}\;\mathrm{activity}\;\left(\%\right)=\;\frac{\left(A_{\mathrm{blank}}-{\;A}_{\mathrm{sample}}\right)\;}{A_{\mathrm{blank}}}\;\times 100$$

EC_50_ values were calculated as described above for DPPH.

### 3.7. Statistical Analysis

All the in vitro assays were performed in triplicate and means of data analyzed using the analysis of variance ANOVA) by SAS program Version 9.0. (SAS Institute, Cary, NC, USA). Statistical significance of differences between samples was accepted at *p* < 0.05 using the Duncan’s multiple range test.

## 4. Conclusions

This research work studied the antioxidant properties of 12 peptide sequences identified from the bromelain digest of mung bean proteins. DRSA was correlated to the presence of cysteine residues at the N-terminal and C-terminal of peptides, which indicate significant contributions of the sulfhydryl groups. In contrast, the presence of phenylalanine had a negative effect on the DRSA of peptides. The presence of tryptophan also reduced antioxidant potency. Scavenging of other free radicals such as hydroxyl and superoxide was also highly dependent on the presence of cysteine in the peptides. The role of glutamine was dependent on location within the peptide with the N-terminal position contributing to stronger antioxidant properties while presence at the penultimate position had an opposite effect. Overall, CTN, CGN, and HC exhibited the strongest free radical scavenging and metal ion chelation properties with values that are comparable to those obtained for GSH.

## Figures and Tables

**Table 1:** Antioxidant activities of selected peptide sequences identified from fractions 1B and 1C *.

Fractions	Sequences	*m*/*z*	z	Mass (Da)	DRSA (EC_50_, mg/mL)	HRSA (EC_50_, mg/mL)	SRSA (EC_50_, mg/mL)	FRAP (mM Fe^2+^ Reduced/mg Peptide)	MCA (EC_50_, mg/mL)
**1B**	HC	130.19	2	258.08	0.29 ± 0.01 ^ef^	0.56 ± 0.03 ^ef^	0.15 ± 0.01 ^ef^	0.71 ± 0.03 ^a^	1.34 ± 0.04 ^b^
	CGN	147.22	2	292.08	0.28 ± 0.01 ^f^	0.74 ± 0.02 ^e^	0.04 ± 0.01 ^g^	0.08 ± 0.00 ^e^	0.04 ± 0.01 ^g^
	LAN	158.95	2	316.18	ND	5.31 ± 0.06 ^b^	1.54 ± 0.02 ^c^	0.01 ± 0.00 ^h^	0.36 ± 0.02 ^defg^
	CTN	169.09	2	336.11	0.30 ± 0.01 ^e^	0.64 ± 0.03 ^ef^	0.04 ± 0.01 ^g^	0.11 ±0.00 ^d^	0.04 ± 0.01 ^g^
	LAF	175.41	2	349.20	ND	0.31 ± 0.03 ^f^	0.02 ± 0.01 ^g^	0.01 ± 0.00 ^h^	10.78 ± 0.89 ^a^
	CSGD	191.13	2	380.10	0.30 ± 0.01 ^e^	0.78 ± 0.03 ^e^	0.04 ± 0.01 ^g^	0.16 ± 0.00 ^c^	0.26 ± 0.02 ^efg^
	MMGW	262.70	2	523.19	1.39 ± 0.01 ^c^	5.96 ± 0.47 ^a^	1.58 ± 0.04 ^b^	0.04 ± 0.00 ^g^	0.77 ± 0.03 ^cd^
	QFAAD	276.32	2	550.24	ND	2.67 ± 0.23 ^c^	0.57 ± 0.01 ^e^	0.01 ± 0.00 ^h^	1.16 ± 0.02 ^bc^
**1C**	ERF	226.09	2	450.22	4.87 ± 0.54 ^a^	2.25 ± 0.33 ^d^	1.34 ± 0.01 ^d^	0.01 ± 0.00 ^h^	0.63 ± 0.01 ^def^
	EYW	249.12	2	496.20	1.32 ± 0.03 ^d^	ND	1.75 ± 0.03 ^a^	0.06 ± 0.00 ^f^	0.69 ± 0.02 ^de^
	FLQL	260.42	2	519.31	ND	ND	ND	0.01 ± 0.00 ^h^	ND
	QFAW	276.17	2	550.25	1.67 ± 0.01 ^b^	ND	ND	0.03 ± 0.00 ^g^	0.07 ± 0.0 1 ^g^
**Glutathione (GSH)**					0.03 ± 0.00 ^e^	0.60 ± 0.01 ^ef^	0.04 ± 0.01 ^g^	0.61 ± 0.02 ^b^	0.18 ± 0.01 ^gf^

* ND: no detectable activity; EC_50_ = effective concentration that scavenged 50% of free radicals (DPPH, SRSA, HRSA) or bind 50% of iron (metal chelation); DRSA, DPPH radical scavenging activity; HRSA, hydroxyl radical scavenging activity; SRSA, superoxide radical scavenging activity; FRAP, ferric reducing antioxidant power; MCA, metal chelating activity. Data are expressed as mean ± standard deviation (*n* = 3). a,b,c,… Different letters indicate significantly different (*p* < 0.05) mean values in the same column.

## Data Availability

Data are contained within the article. The peptide sequences have been submitted to the BIOPEP database (http://www.uwm.edu.pl/biochemia/index.php/en/biopep).

## Sample Availability

Samples of the compounds are not available from the authors.
